# Can Visceral Adiposity Index Serve as a Simple Tool for Identifying Individuals with Insulin Resistance in Daily Clinical Practice?

**DOI:** 10.3390/medicina55090545

**Published:** 2019-08-29

**Authors:** Ladislav Štěpánek, Dagmar Horáková, Ľubica Cibičková, Helena Vaverková, David Karásek, Marie Nakládalová, Jana Zapletalová

**Affiliations:** 1 Department of Public Health, Faculty of Medicine and Dentistry, Palacký University Olomouc, 77515 Olomouc, Czech Republic; 2 3rd Department of Internal Medicine—Nephrology, Rheumatology and Endocrinology, University Hospital Olomouc and Faculty of Medicine and Dentistry, Palacký University Olomouc, 77900 Olomouc, Czech Republic; 3 Department of Occupational Medicine, University Hospital Olomouc and Faculty of Medicine and Dentistry, Palacký University Olomouc, 77900 Olomouc, Czech Republic; 4 Department of Biophysics, Faculty of Medicine and Dentistry, Palacký University Olomouc, 77515 Olomouc, Czech Republic

**Keywords:** visceral adiposity index, homeostasis model assessment of insulin resistance, metabolic syndrome, cardiometabolic risk

## Abstract

*Background and objectives:* The visceral adiposity index (VAI), estimating visceral adiposity dysfunction through a simple formula, could serve as a useful tool for identifying individuals at higher cardiometabolic risk. Its relationship with insulin resistance (IR), assessed using the homeostasis model assessment of IR (HOMA-IR), and metabolic syndrome (MetS) components remains unclear. The study aimed to investigate the association of VAI with both HOMA-IR and MetS. *Materials and Methods:* After undergoing anthropometric and biochemical studies, 783 individuals were divided into three groups according to a number of present MetS components. The VAI cut-offs signaling MetS and HOMA-IR were determined by maximizing the sum of the sensitivity and specificity. Correlation analysis was performed to explore the associations between VAI and other tested parameters. A logistic stepwise regression analysis was applied to identify statistically significant determinants of HOMA-IR. Given the variability of reference values, two thresholds of HOMA-IR were applied, namely 2.0 and 3.8. *Results:* VAI increased significantly between the groups with a rising number of MetS components. The VAI cut-off for MetS was 2.37, with a sensitivity of 0.86 and a specificity of 0.78. The same cut-off point identified subjects with HOMA-IR = 3.8, with a sensitivity of 0.79 and a specificity of 0.66. The VAI cut-off for HOMA-IR = 2.0 was 1.89, with a sensitivity of 0.74 and a specificity of 0.68. The strongest correlations of VAI were noted with HOMA-IR (r = 0.51) and insulin (r = 0.49), respectively, while the strongest correlation of HOMA-IR was with waist circumference (r = 0.54). Not one of the routine parameters was a significant predictor in the regression analysis. *Conclusions:* The obtained results show an existing association of VAI with HOMA-IR. The high sensitivity and specificity of the cut-offs may allow the application of VAI in common clinical practice.

## 1. Introduction

Cardiometabolic diseases are the global leading causes of death and represent a significant economic burden on health systems. The diseases are largely preventable and, given the current epidemiological situation, the pressure to search for and use new effective preventive tools increases. Metabolic syndrome (MetS) has been defined as a single nosological entity, among others, to clearly identify individuals with high cardiometabolic risk eligible for targeted preventive interventions [1,2]. MetS is a cluster of clinical, metabolic, and biochemical abnormalities, such as central adiposity, hypertension, hyperglycemia, and dyslipidemias. According to some definitions, visceral obesity, as measured by waist circumference (WC), is stressed over other components of MetS [3]. The prevalence of MetS keeps growing with the rising rates of obesity worldwide, no matter what diagnostic criteria are being used. Although research has been carried out in recent decades on MetS, the exact underlying etiology is still not completely understood [4].

When central obesity is slowly being developed, it is observed that hyperinsulinemia and hyperglycemia also progress slowly in the postprandial state. In the state of obesity, most subjects already present insulin resistance (IR) and hyperinsulinemia, probably the first step of a dysfunctional metabolic system [1]. In a minor part of the obese, however, excessive body fat accumulation does not lead to adverse metabolic effects. Such a condition is referred to as metabolically healthy obesity (MHO). Until now, there have been several criteria of MHO, some of which include IR [5,6,7]. Many other contributing factors and mechanisms of MetS, except for IR, have been proposed, such as adipose tissue dysfunction, chronic inflammation, oxidative stress, circadian disruption, microbiota, genetic factors, and maternal programming, etc. [4].

A method for detecting IR that is easy to use in common clinical practice, albeit still rather expensive for widespread use in primary care, is the homeostasis model assessment of IR (HOMA-IR). Its satisfactory correlation with the most accurate glucose clamp techniques has been confirmed by numerous studies [8].

Recently, there has been an increase in information in the literature about a relatively new predictive model called visceral adiposity index (VAI) that seems to be a reliable indicator of visceral adipose dysfunction; its increase is strongly associated with cardiometabolic risk. The VAI calculation is based on the routine anthropometric and biochemical parameters that are part of preventive examinations in primary care [9].

The precise relationship of VAI and HOMA-IR remains unclear. For its simple determination, VAI could serve as a very easy indicator of early metabolic dysfunction occurring long before the development of cardiometabolic diseases. The study aimed to assess the relationship of VAI with both MetS and HOMA-IR, determine VAI cut-off points, and to assess the potential of the application of VAI in common clinical practice.

## 2. Material and Methods

### 2.1. Study Subjects

Between March 2010 and November 2018, individuals eligible for the study were selected during their first visits at two cooperating medical offices: a general practitioner office in the Pardubice Region and an outpatient center of the 3rd Department of Internal Medicine, University Hospital Olomouc, Czech Republic. None of the included subjects was treated with oral antidiabetic drugs or insulin. The study comprised 783 individuals (426 males and 357 females) with a mean age of 46 years. In these subjects, the below laboratory analyses were performed and, at the same time, their basic anthropometric parameters (height, weight, WC) and blood pressure (BP), as a mean of three resting recordings at a single visit, were measured. The obtained data were used to calculate body mass index (BMI), HOMA-IR, VAI, and atherogenic index of plasma (AIP) for each participant. Based on their results, all subjects were divided into three groups according to a number of MetS components met: Group A (none or one component), Group B (two components), and Group C (at least three components = subjects with MetS). The components come from the International Diabetes Federation (IDF) criteria composing the following features: Abdominal obesity (WC ≥ 94 cm in men and ≥ 80 cm in women), fasting glucose (FG) ≥ 5.6 mmol/L or previous diagnosis of type 2 diabetes mellitus (T2DM), BP ≥ 130/85 mmHg and/or hypertension under medication, triglycerides (TG) ≥ 1.7 mmol/L or hypertriglyceridemia under drug treatment, and high-density lipoprotein cholesterol (HDL-C) < 1.0 mmol/L in men and <1.3 mmol/L in women [10].

The study was conducted according to the principles stated in the Declaration of Helsinki. The study was approved by the Local Ethics Committee of the University Hospital, Olomouc (Approval No.: 147/16). To be included in the study, all subjects signed informed consent forms after they were explained all information regarding the study.

### 2.2. Laboratory Analysis

All laboratories participating in the study meet the same national accreditation. In all cases, the principles of proper laboratory practice were followed and the laboratories were under systematic intra- and inter- laboratory control of the accuracy of examinations. Venous blood was always sampled in the morning, after 12-h fasting. The following biochemistry parameters were analyzed on Cobas 8000 (Roche Diagnostics GmbH, Manheim, Germany) using the fresh serum on the day of blood sampling: FG, total cholesterol (TC), low-density lipoprotein cholesterol (LDL-C), HDL-C, TG, apolipoprotein B (ApoB). TC, TG and HDL-C were determined enzymatically. LDL-C was calculated using Friedewald formula. Glucose was determined using hexokinase method (Roche, Basel, Switzerland). Concentration of ApoB was determined immunoturbidimetrically (TinaQuant Apo B kits, Roche, Mannheim, Germany). To determine serum insulin concentrations, serum was deep frozen within no more than 2 h from blood sampling. The separated serum was stored at −80°C until the day of analysis (for not later than one week). The insulin concentration analysis itself was carried out using chemiluminescent microparticle immunoassay on Architect i1000SR (Abbott Laboratories, Chicago, IL, USA).

### 2.3. Statistical Analysis

Statistical analyses were conducted with the IBM SPSS Statistics, version 22 (SPSS Inc, Chicago, IL, USA). All numerical variables were characterized with descriptive statistics. The Kruskal–Wallis test and Dunn’s post hoc test were used to compare the study groups. A *p* value of less than 0.05 indicates statistical significance. The Shapiro–Wilk test was used to test data distribution. Because of the dominating right-skewed data distribution, correlations of selected variables were quantified with Spearman’s correlation coefficient (r) and the level of significance (*p*) was determined. VAI cut-offs were determined using the maximized sum of sensitivity and specificity. Sensitivity and specificity were considered equally important and, therefore, their relative weights in the sum were 1:1. In other words, we searched for the maximum of Youden’s index as a function of sensitivity and specificity [11]. The accuracy and predictive ability of the cut-offs were verified by calculating the area under the receiver operating characteristic curves (AUC). A logistic stepwise forward regression analysis was applied to identify the statistically significant determinants of HOMA-IR. Since there is not an agreement on the threshold of HOMA-IR signaling established IR, we applied both maximum and minimum values identified in available population-based studies of various European nations, namely 2.0 and 3.8 [12,13]. The following formulas were used to calculate the aforementioned indices [8,9,14]. Units of the variables are given in Table 1:

$$\begin{array}{c} HOMA\text{-}IR=\frac{FG\times insulin}{22.5}\\ AIP=log\frac{TG}{HDL}\\ VAI\;in\;males=\left(\frac{WC}{39.68+\left(1.88\times BMI\right)}\right)\times \left(\frac{TG}{1.03}\right)\times \left(\frac{1.31}{HDL\;cholesterol}\right)\\ VAI\;in\;females=\left(\frac{WC}{36.58+\left(1.89\times BMI\right)}\right)\times \left(\frac{TG}{0.81}\right)\times \left(\frac{1.52}{HDL\;cholesterol}\right) \end{array}$$

## 3. Results

### 3.1. Characteristics of the Study Population

The basic characteristics of all subjects with respect to a study group are shown in Table 1. It is apparent that mean values of all metabolic parameters deteriorated with a rising number of present MetS components. Accordingly, the mean values in Group A were normal or borderline, contrasting with the abnormal values in Group C. All differences reached statistical significance except for TC and LDL-C between Group B and Group C. According to BMI and WC, Group A consisted of normal weight subjects, while Group C consisted of subjects in the obesity range. The mean age significantly increased from Group A to Group C. The last lines of Table 1 demonstrate significantly growing mean values of both HOMA-IR and VAI from Group A to Group C. The mean value of HOMA-IR in Group A was far below the aforementioned lower threshold of HOMA-IR, whereas the mean value in Group C was above the upper threshold.

### 3.2. VAI Cut-Offs

All identified cut-offs are shown in Table 2. The VAI cut-off separating subjects meeting the MetS definition (Group C) from the others (Group A and Group B), regardless of sex, was 2.37, with a sensitivity of 0.86 and a specificity of 0.78. The AUC was 0,878 (95% confidence interval (CI) 0.853–0.903) for MetS diagnosing. The VAI cut-off for the lower threshold of HOMA-IR was 1.89 and 2.37 for the upper threshold, respectively. The sum of sensitivity and specificity for detecting HOMA-IR was similar for both thresholds but generally lower than in case of the previous MetS cut-off. The same applies to the AUC (Table 3). High negative predictive values were reported for all cut-offs, which supports their good specificity.

### 3.3. Correlation Analysis

**Figure 1:**
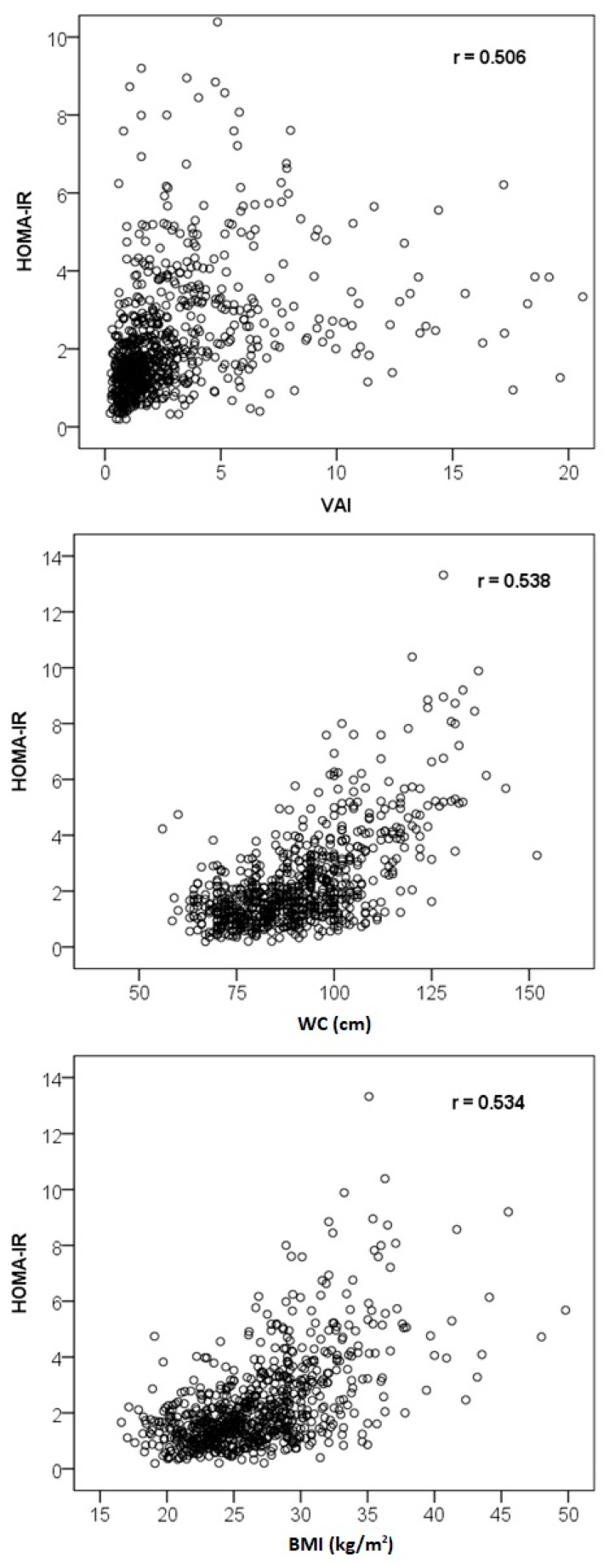
Scatter diagrams depicting correlations of homeostasis model assessment of insulin resistance (HOMA-IR) with visceral adiposity index (VAI), waist circumference (WC) and body mass index (BMI).

Table 3 shows Spearman’s correlation coefficients between investigated indices and other tested parameters. Leaving out parameters included in the VAI formula, the highest correlation coefficients of VAI were noted with HOMA-IR (r = 0.51) and insulin (r = 0.49), respectively. The correlation between VAI and FG was weaker (r = 0.31). The strongest correlation of FG was recorded with WC (r = 0.48). Unsurprisingly, there was a very strong correlation between VAI and AIP (r = 0.98) due to the inclusion of the same variables in their formulas. The strongest correlations of HOMA-IR were noted with WC (r = 0.54), BMI (r = 0.53), AIP (r = 0.49), TG (r = 0.44), and HDL-C (r = −0.43). All these moderate correlations reached high statistical significance. In other words, HOMA-IR was more correlated with both WC and BMI than with VAI (Figure 1). There were no other moderate or strong correlations between any tested parameters in the entire sample, except for few strong associations among lipid parameters, the strongest being correlations of TC with LDL-C (r = 0.83) and ApoB (r = 0.81), respectively.

### 3.4. Regression Analysis—Prediction of HOMA-IR

Logistic stepwise regression for HOMA-IR prediction (dependent variable) was used with respect to all potential predictors (characteristics) listed in Table 1. No significant predictor of the lower or upper HOMA-IR threshold was identified except for the obvious predictors: FG and insulin.

## 4. Discussion

The obtained results of the correlation analysis confirm an association of VAI and HOMA-IR, although the logistic regression did not prove VAI a stronger predictor of HOMA-IR among others. The value of VAI increases significantly with a growing number of present MetS components; thus, demonstrating the ability of VAI to reflect a level of the metabolic disorder connected with MetS. The VAI cut-off point identifying MetS subjects was 2.37 in our study, with a sensitivity of 0.86 and a specificity of 0.78. In a study of 92 overweight and obese patients conducted by Pekgor et al., the VAI cut-off identifying MetS subjects (the same IDF criteria of MetS) was 2.21. Both sensitivity (0.76) and specificity (0.69) differed by approximately 10% from our results. However, an agreement between the studies is lower in the determination of the VAI cut-off signaling IR, which was set at 2.31 by Pekgor et al., with a sensitivity of 0.69 and a specificity of 0.64. However, it must be noted that the authors used a different threshold of HOMA-IR than we did (HOMA-IR = 2.5 by Pekgor et al.). In our study, the VAI cut-offs were 1.89 for HOMA-IR = 2.0 and 2.37 for HOMA-IR = 3.8, respectively, with a sensitivity and a specificity only slightly higher on both HOMA-IR thresholds in comparison with the study by Pekgor et al. [15]. Except that the situation is complicated by the absence of a unified HOMA-IR threshold, the high and similar sensitivity and specificity values in both studies indicate the potential of VAI to detect different levels of HOMA-IR. In the present study, especially the negative predictive values were remarkable. Around 95% of the study subjects with the value of VAI below 2.37 neither met MetS criteria nor had HOMA-IR above 3.8.

A study by Amato et al. conducted in 1764 primary care patients determined the VAI cut-offs for separating those with MetS (the NCEP-ATP III criteria) with respect to age quintiles. For individuals at the age of ≥42 and <52 years, as approximately in our study, the cut-off was 1.92, with a sensitivity of 0.91 and a specificity of 0.73. Higher cut-off points, similar to those in the present study and in the study by Pekgor et al. were obtained at lower ages (around 30 years) [15,16]. Amato et al. in another study including 91 patients with T2DM defined the value of VAI signaling T2DM as 2.0. Its sensitivity (0.65) was lower than in the previous study setting the VAI cut-off for MetS; however, the specificity (0.81) was higher. In patients with T2DM, Amato et al. emphasize the presence of adipose tissue dysfunction resulting in increased VAI. What is more, the authors also show significant correlations of VAI and some adipocytokine levels [9]. In a study of 2754 community-dwelling people by Liu et al., among all common obesity indices, VAI was the only index significantly associated with both prediabetes and T2DM in both sexes after adjusting for potential confounders in a logistic regression model [17].

Correlations between VAI and glucose metabolism parameters were demonstrated in Table 3, with correlation coefficients of VAI being the highest with HOMA-IR (r = 0.51) and insulin (r = 0.49), respectively. The correlation of VAI with FG was weaker (r = 0.31). The same trend with the correlations of VAI being the strongest with HOMA-IR and insulin, respectively, were reported in the study by Amato et al. among the T2DM patients (r = 0.32 and r = 0.33, respectively) and in a study by Jabłonowska-Lietz et al. in 106 obese subjects (r = 0.46 and r = 0.44, respectively). In these two studies, the correlation coefficients between VAI and FG were only 0.03 and 0.37, respectively [9,18]. This trend could be partly explained by the considerable FG variability, commonly observed especially in individuals with glucose metabolism disorders [19].

VAI has been proven to be primarily an indicator of adipose distribution and function that indirectly expresses cardiometabolic risk. Both adipose tissue dysfunction and IR are key pathogenesis mechanisms of MetS, and therefore cardiometabolic diseases. This is confirmed by the significant correlation of VAI, reflecting the adipose dysfunction, with HOMA-IR, reflecting IR. The study by Jabłonowska-Lietz et al. showed a significant correlation between VAI and the visceral adipose tissue (VAT) index measured by bioelectric impedance. VAT is strongly associated with the risk of cardiovascular diseases and metabolic disorders. Therefore, VAI was proposed as a surrogate marker of VAT and a useful tool in daily clinical practice and in population studies for the assessment of cardiometabolic risk associated with visceral adiposity [17].

Several studies suggested the ability of VAI to detect and assess various forms of atherosclerosis. A study by Li et al. showed a close association of VAI with an increased risk of intracranial atherosclerotic stenosis in 450 middle-aged and elderly Chinese females [20]. Randrianarisoa et al. in a study of 731 middle-aged adults proved a correlation of VAI with carotid intima-media thickness independently of other established cardiovascular risk factors. After adjusting for other covariates including age, systolic and diastolic BP, smoking, and high sensitivity C-reactive protein in a multivariate regression analysis, VAI was found to be an independent determinant of HOMA-IR [21]. A study by Biswas et al. in a total of 200 patients with acute coronary syndrome showed that VAI was a good predictor of clinical and coronary angiographic severity based on logistic regression analysis. Given the simplicity of VAI determination, this index could serve as a useful tool for the detection of patients at risk of both metabolic and atherosclerotic complications [22].

As seen from their formulas, VAI includes the same parameters as AIP, however, the correlations of VAI with HOMA-IR were slightly stronger than those of AIP. Therefore, the inclusion of more parameters in the VAI formula gives it higher predictive abilities. There are also studies supporting relationship of the simultaneous presence of elevated serum TG and increased WC (so-called hypertriglyceridemic waist) with the level of IR [23].

In recent years there have been studies questioning the concept of MHO. They show that, even when metabolic health is maintained, obesity remains a risk factor for cardiovascular disease and metabolic health, and a large proportion of individuals with MHO converts to an unhealthy phenotype over time [5,6]. As already mentioned, some criteria of MHO include HOMA-IR. Liu et al. in their study applied five different criteria of MHO in 4757 random adults to find the lowest prevalence of MHO (4.2%) when applying a criterion that included HOMA-IR (≤1.95) [7]. Also in adolescents, MHO prevalence decreases when IR is part of the criteria [24]. Khawaja et al. in their study identified a definite disparity in cardiovascular risk between two groups of obese individuals-either with or without IR, along with a correspondingly unfavorable metabolic profile in the latter [25]. Overall, IR is an important part of the obesity phenotype and plays a role in obesity outcomes. VAI may be a useful tool to assess the obesity phenotype also with respect to its association with IR. Conclusive findings on the differences in outcomes of the obesity phenotypes can come from long-term prospective studies.

The study has certain limitations. One is the fact that it used laboratory data obtained by single measurement, causing possible bias due to the natural intra-individual variability of the analyzed parameters, as is well known, for instance, in fasting glucose. Another limitation is that common long-term antihypertensive and hypolipidemic therapy was present in some subjects of the study population, affecting plasma lipid levels and BP values. In particular, the prevalence of the antihypertensive and hypolipidemic therapy was 17% and 14% in Group A, 23% and 16% in Group B, and 25% and 22% in Group C, respectively.

## 5. Conclusions

Given the simplicity of WC and BMI measurement and TG and HDL-C determination, we suggest that VAI could be an easy tool for the evaluation of adipose tissue dysfunction and its associated cardiometabolic risk in various patients, mainly in the absence of an overt MetS. VAI can be calculated automatically by medical software. The high sensitivity and specificity of the obtained cut-offs enable the widespread use of VAI in common clinical practice to identify individuals with high cardiometabolic risk eligible for targeted preventive interventions. However, both WC and BMI have a significant relationship with HOMA-IR. These results are valid for middle-aged European adults.

## Figures and Tables

**Table 1:** Basic metabolic and clinical characteristics in study groups. Statistical significance (*p*) of differences between the study groups was <0.001 unless otherwise indicated.

Characteristics	Group A	Group B	Group C
	Mean ± SD (Median)	Mean ± SD (Median)	Mean ± SD (Median)
n	256 (F164, M92)	301 (F153, M148)	226 (F109, M117)
Age (years)	41.4 ± 14.6 (39.0)	46.0 ± 14.5 (47.0)	52.8 ± 12.1 (54.0)
Glucose (mmol/L)	4.78 ± 0.44 (4.80)	5.11 ± 0.66 (5.07)	5.92 ± 1.24 (5.80)
Insulin (mIU/L)	6.53 ± 3.45 (6.00)	8.29 ± 4.40 (7.40)	15.26 ± 10.96 (13.70)
Total cholesterol (mmol/L) *	5.87 ± 1.39 (5.81)	6.34 ± 1.49 (6.34)	6.67 ± 1.92 (6.33)
HDL cholesterol (mmol/L)	1.78 ± 0.43 (1.71)	1.41 ± 0.37 (1.35)	1.14 ± 0.34 (1.09)
LDL cholesterol (mmol/L) **	3.58 ± 1.32 (3.47)	3.87 ± 1.32 (3.78)	3.89 ± 1.60 (3.76)
Triglycerides (mmol/L)	1.10 ± 0.38 (1.09)	2.48 ± 2.49 (1.86)	4.31 ± 4.67 (2.70)
Apolipoprotein B (g/L)	1.05 ± 0.32 (1.03)	1.21 ± 0.34 (1.19)	1.31 ± 0.40 (1.26)
Waist circumference (cm)	79.2 ± 9.2 (79.0)	89.0 ± 12.0 (90.0)	106.2 ± 14.2 (104.0)
BMI (kg/m²)	23.5 ± 2.9 (23.2)	26.2 ± 3.8 (26.0)	31.2 ± 4.4 (30.6)
HOMA-IR	1.40 ± 0.77 (1.26)	1.89 ± 1.06 (1.68)	4.07 ± 3.35 (3.42)
VAI	1.04 ± 0.46 (1.01)	3.15 ± 5.02 (2.10)	7.17 ± 9.35 (4.25)
AIP	−0.23 ± 0.21 (−0.36)	0.16 ± 0.33 (−0.03)	0.47 ± 0.37 (0.23)

SD, standard deviation; F, female; M, male; HDL, high-density lipoprotein; LDL, low-density lipoprotein; BMI, body mass index; HOMA-IR, homeostasis model assessment of insulin resistance; VAI, visceral adiposity index; AIP, atherogenic index of plasma. * *p* (Group A vs. Group B) = 0.002, *p* (Group B vs. Group C) = 0.612; ** *p* (Group A vs. Group B) = 0.029, *p* (Group B vs. Group C) = 1.

**Table 2:** VAI cut-offs maximizing the sum of sensitivity and specificity for the diagnosis of metabolic syndrome or HOMA-IR.

Target Parameter	VAI Cut-Off	Sensitivity	Specificity	PPV	NPV	AUC (95% CI)
Metabolic syndrome	2.372	0.863	0.781	0.615	0.933	0.878 (0.853–0.903)
HOMA-IR = 2.0	1.894	0.738	0.684	0.634	0.779	0.770 (0.735–0.804)
HOMA-IR = 3.8	2.372	0.785	0.662	0.294	0.945	0.765 (0.721–0.808)

VAI, visceral adiposity index; HOMA-IR, homeostasis model assessment of insulin resistance; PPV, positive predictive value; NPV, negative predictive value, AUC, area under the receiver operating characteristic curve; CI, confidence interval.

**Table 3:** Spearman’s correlation coefficients (r) between several investigated indices and other tested parameters with stated statistical significance (*p*).

Characteristics	VAI	Glucose	Insulin	HOMA-IR
Age	0.171 **	0.346 **	0.081 *	0.150 **
Glucose	0.313 **	–	0.359 **	0.528 **
Insulin	0.485 **	0.359 **	–	0.978 **
Total cholesterol	0.304 **	0.083 *	0.039	0.047
HDL cholesterol	−0.730 **	−0.252 **	−0.410 **	−0.426 **
LDL cholesterol	0.136 **	0.065	−0.012	−0.005
Triglycerides	0.938 **	0.273 **	0.430 **	0.444 **
Apolipoprotein B	0.385 **	0.134 **	0.132 **	0.150 **
Waist circumference	0.535 **	0.477 **	0.487 **	0.538 **
BMI	0.496 **	0.449 **	0.490 **	0.534 **
VAI	–	0.313 **	0.485 **	0.506 **
AIP	0.975 **	0.300 **	0.470 **	0.488 **

VAI, visceral adiposity index; HOMA-IR, homeostasis model assessment of insulin resistance; HDL, high-density lipoprotein; LDL, low-density lipoprotein; VAI, visceral adiposity index; AIP, atherogenic index of plasma. * *p* < 0.05; ** *p* < 0.01.
